# Supplementary material for: Integrative genomic and transcriptomic analysis of leiomyosarcoma
Source: Nat Commun. 2018 Jan 10;9:144. doi: 10.1038/s41467-017-02602-0 (PMC5762758; doi:10.1038/s41467-017-02602-0)
Supplement: Supplementary file 1 — Supplementary Information [file 41467_2017_2602_MOESM1_ESM.pdf]

Supplementary Information

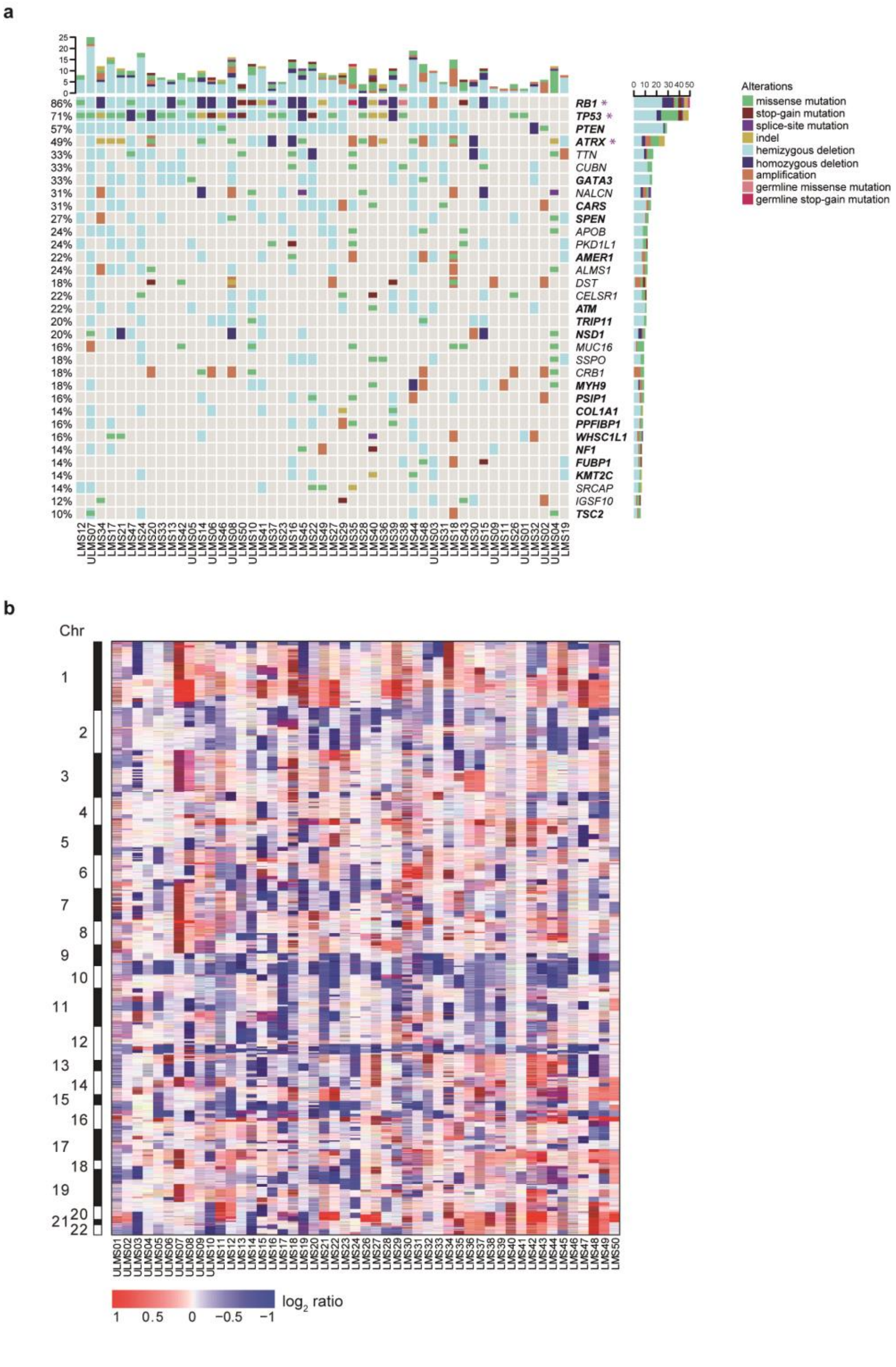

Supplementary Figure 1 | Genomic imbalances in adult LMS.

a Genomic imbalances affecting frequently mutated genes in adult LMS. Rows represent individual genes, columns represent individual tumors. Genes are sorted according to frequency of SNVs, indels, and CNAs (left). Asterisks indicate significantly mutated genes according to MutSigCV. Bars depict the number of alterations for individual tumors (top) and genes (right). Established cancer genes are shown in bold. Types of mutations are annotated according to the color code. **b** Heatmap of genomic gains (red) and losses (blue) for each tumor (horizontal axis) by chromosomal location (vertical axis). Chr, chromosome.

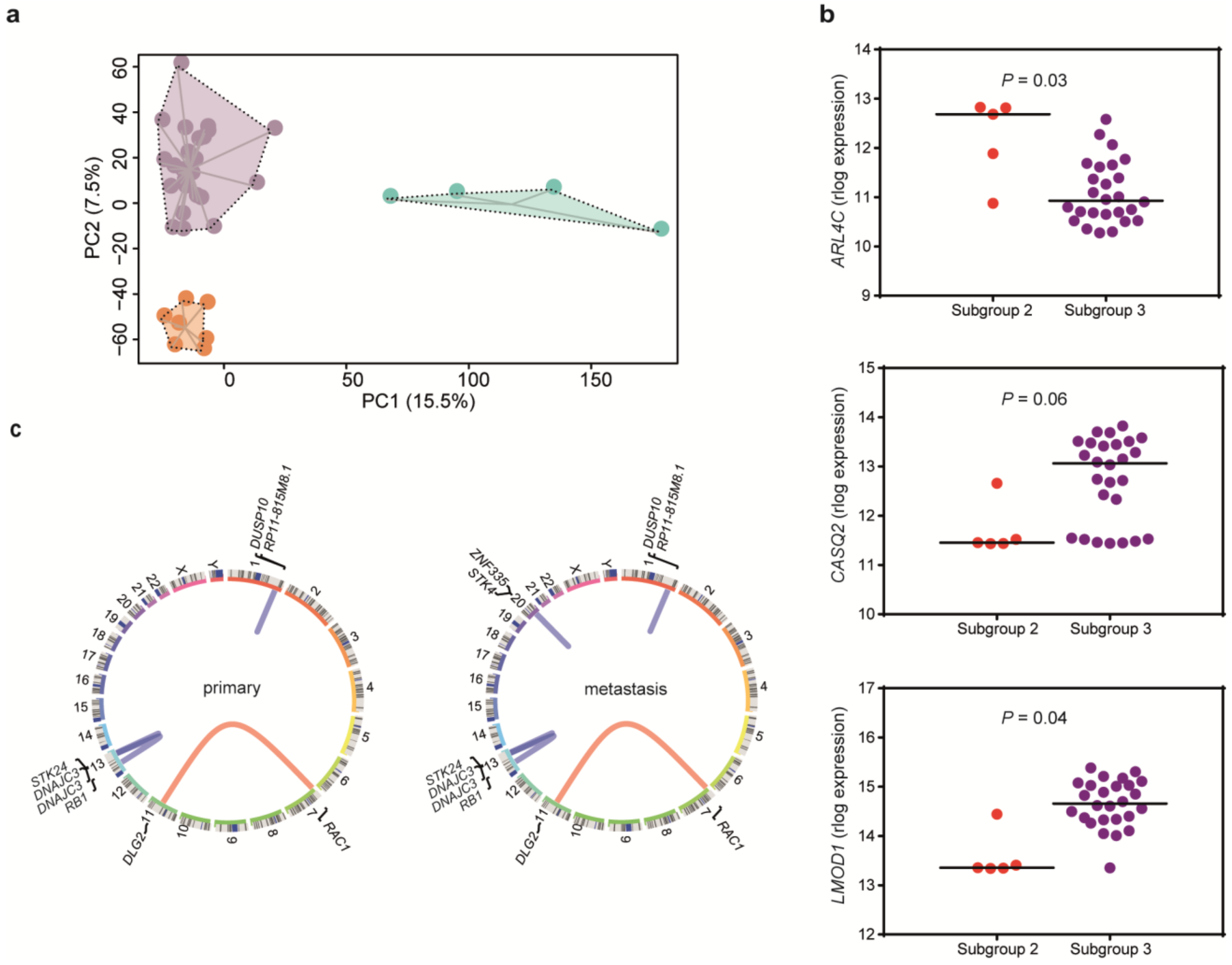

Supplementary Figure 2 | Transcriptomic characterization of adult LMS.
a Principal component (PC) analysis of gene expression profiles from 37 tumors showing separation into three distinct clusters according to values for PC1 (variance, 15.5%; horizontal axis) and PC2 (variance, 7.5%; vertical axis). **b** Column scatter plots showing expression of *ARL4C*, *CASQ2,* and *LMOD1* in LMS subgroup 2 and 3 samples. Statistical significance was assessed using an unpaired *t*-test. **c** Structural variant plots of fusion transcripts detected in a primary LMS tumor (left) and a corresponding metastasis (right).

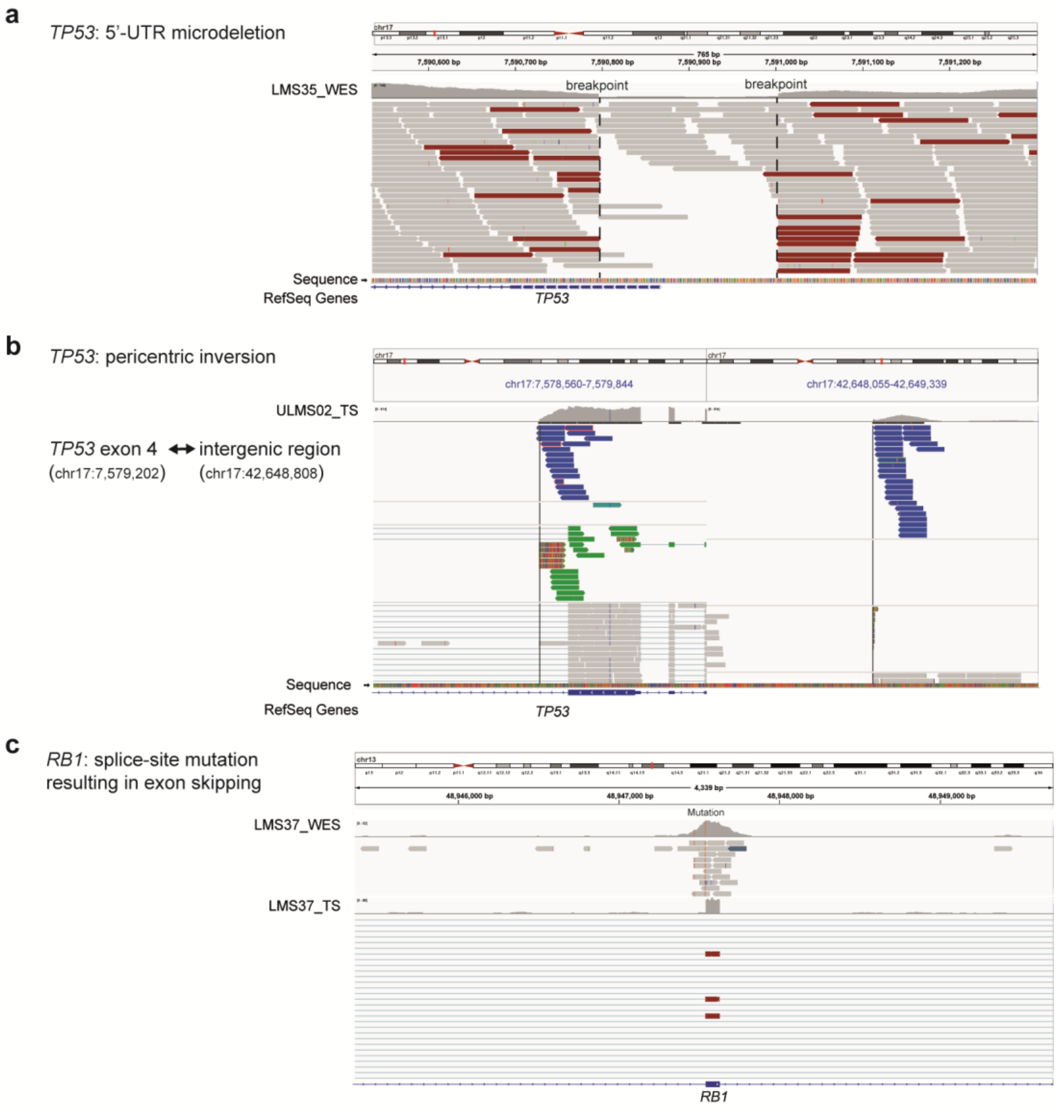

Supplementary Figure 3 | Genetic lesions targeting *TP53* and *RB1* in adult LMS.

a Microdeletion affecting the *TP53* transcription start site in case LMS35. **b** Pericentric inversion of chromosome 17 disrupting *TP53* in case ULMS02. **c** *RB1* splice-site mutation resulting in exon skipping in case LMS37. UTR, untranslated region; WES, whole-exome sequencing; TS, transcriptome sequencing.

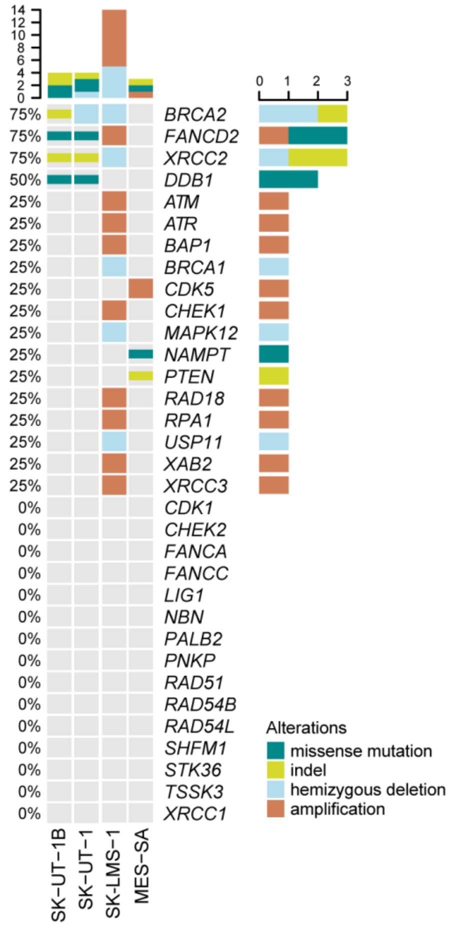

Supplementary Figure 4 | Whole-genome sequencing of LMS cell lines identifies alterations in genes reported to be synthetic lethal to PARP inhibition.

Rows represent individual genes, columns represent individual cell lines. Genes are sorted according to frequency of SNVs, indels, and CNAs (left). Bars depict the number alterations for individual cell lines (top) and genes (right). Types of alterations are annotated according to the color code (bottom).
