# Supplementary material for: Integrative genomic and transcriptomic analysis of leiomyosarcoma
Source: Nat Commun. 2018 Jan 10;9:144. doi: 10.1038/s41467-017-02602-0 (PMC5762758; doi:10.1038/s41467-017-02602-0)
Supplement: Supplementary file 3 — Description of Additional Supplementary Files [file 41467_2017_2602_MOESM3_ESM.pdf]

Description of Supplementary Files

File Name: Supplementary Data 1
Description: Clinical characteristics and molecular analysis of 49 LMS patients.
